# Gut microbiome and metabolites: The potential key roles in pulmonary fibrosis

**DOI:** 10.3389/fmicb.2022.943791

**Published:** 2022-10-06

**Authors:** Yinlan Wu, Yanhong Li, Yubin Luo, Yu Zhou, Ji Wen, Lu Chen, Xiuping Liang, Tong Wu, Chunyu Tan, Yi Liu

**Affiliations:** ^1^Department of Rheumatology and Immunology, West China Hospital, Sichuan University, Chengdu, China; ^2^Rare Diseases Center, West China Hospital, Sichuan University, Chengdu, China; ^3^Institute of Immunology and Inflammation, Frontiers Science Center for Disease-Related Molecular Network, West China Hospital, Chengdu, China; ^4^Department of Respiratory and Critical Care Medicine, Chengdu First People’s Hospital, Chengdu, China

**Keywords:** gut microbiome, metabolites, pulmonary fibrosis, gut–lung axis, short chain fatty acid, amino acid

## Abstract

There are a wide variety of microbiomes in the human body, most of which exist in the gastrointestinal tract. Microbiomes and metabolites interact with the host to influence health. Rapid progress has been made in the study of its relationship with abenteric organs, especially lung diseases, and the concept the of “gut–lung axis” has emerged. In recent years, with the in-depth study of the “gut–lung axis,” it has been found that changes of the gut microbiome and metabolites are related to fibrotic interstitial lung disease. Understanding their effects on pulmonary fibrosis is expected to provide new possibilities for the prevention, diagnosis and even treatment of pulmonary fibrosis. In this review, we focused on fibrotic interstitial lung disease, summarized the changes the gut microbiome and several metabolites of the gut microbiome in different types of pulmonary fibrosis, and discussed their contributions to the occurrence and development of pulmonary fibrosis.

## Introduction

Interstitial lung disease (ILD) is an umbrella term characterized by chronic lung inflammation and elevated levels of chronic inflammatory cells with varying degrees of pulmonary fibrosis (Raghu et al., 2018). Patients with ILD have a poor prognosis and poor response to treatment due to pulmonary fibrotic lesions (Kalchiem-Dekel et al., 2018). ILD can be caused by connective tissue disease, radiation damage, particle inhalation and other reasons, but cases have no clear etiology, which is called idiopathic pulmonary fibrosis (IPF) (Kolb and Vašáková, 2019). Although there are many studies on pulmonary fibrosis, the exact mechanism of its pathogenesis and development is still not completely clear.

The human body contains a variety of microorganisms, including bacteria, fungi, viruses, archaea and protozoa (Lynch and Pedersen, 2016), most of which are found in the gastrointestinal tract (Marsland et al., 2015). The various microbial communities that colonize the gut of the host are called the gut microbiota (Kc et al., 2020). With in-depth, comprehensive analyses of the microbiome and metabolome, it has been gradually found that metabolites and antigens of the gut microbiome may regulate the host (Cruz et al., 2021). The intestinal microbiome could affect the occurrence, progression and prognosis of diseases in various ways through bidirectional flow between the lung and intestine via the blood and lymphatic systems, such as inflammation, metabolism and cell signal transduction of microbial metabolites (Marsland et al., 2015; Dickson et al., 2018; Chioma et al., 2021). Thus, the concept of the “gut–lung axis” was proposed and gradually applied to study the pathogenesis and progression of various pulmonary diseases. For example, it has been reported that the dysbiosis of the gut microbiome in systemic sclerosis patients with ILD is more obvious and appears in the early stage of pulmonary fibrosis (Andréasson et al., 2016); It has also been demonstrated that the intestinal metabolite arginine is involved in collagen deposition in IPF patients (Wang et al., 2021). With the development of omics technology and bioinformatics analysis, many studies related to the “gut–lung axis” have been published recently, but there is no summary article on pulmonary fibrosis at present. In this review, we will summarize the alterations of the gut microbiota by different types of pulmonary fibrosis, and the contribution of several of the most well-known intestinal metabolites in the process of pulmonary fibrosis.

## Pathophysiology of interstitial lung disease

Many studies have pointed out that epithelial cells, myofibroblasts and the immune system play a major role in the progression of ILD under the influence of genetic factors, epigenetic reprogramming and environmental factors (Milara et al., 2018; Kadota et al., 2020; Valenzi et al., 2021). (1) Proliferation, apoptosis, senescence and epithelial-mesenchymal transition (EMT) occur in lung epithelial cells due to chronic inflammation, peroxidation stimulation, or gene expression and modification changes, thus participating in pulmonary fibrosis (Smirnova et al., 2016; Xu et al., 2016; Jablonski et al., 2017). Moreover, epithelial cells can secrete transforming growth factor-β (TGF-β), tumor necrosis factor (TNF), a variety of matrix metalloproteinases and chemokines to promote the expression of myofibroblasts, leading to the remodeling of the extracellular matrix (ECM) (King et al., 2011; Selman and Pardo, 2014). (2) In the process of pulmonary fibrosis, a large number of immune cells accumulate and release inflammatory factors (Wynn and Vannella, 2016; Gao et al., 2019). There are two subtypes of macrophages, namely classically activated macrophages (M1) and alternatively activated of macrophages (M2). M1 macrophages produce proinflammatory cytokines, such as TNFα, interleukin-1 (IL-1) and IL-6, to maintain chronic inflammation (Mills, 2012). M2 macrophages secrete a variety of growth factors, including TGF-β and fibroblast growth factor, which contribute to over-repair (Duru et al., 2016). Neutrophil aggregation may participate in amplified tissue remodeling in lung injury through the release of proinflammatory cytokines and the generation of reactive oxygen species (Mayadas et al., 2014). IL-17 secreted by helper T lymphocyte type 17 (Th17) cells promotes lung fibroblast proliferation, resulting in increased type I collagen synthesis and TGF-β and IL-6 expression (Lei et al., 2016). In several clinical and animal studies, there have been controversial results regarding the effects of regulatory cells (Tregs) on lung fibrosis (Reilkoff et al., 2013; Kamio et al., 2018), possibly due to a shift from a protective to a destructive phenotype of Tregs during inflammation (Boveda-Ruiz et al., 2013; Birjandi et al., 2016). (3) Fibroblasts, pericytes and mesenchymal progenitors differentiate into myofibroblasts, leading to lung pathologic excess, and thereby promoting pulmonary fibrosis (Martinez et al., 2017). Myofibroblasts are involved in the accumulation of ECM components, including collagen, fibronectin, tenascin and proteoglycan (Klingberg et al., 2013). The ECM promotes fibroblast differentiation through genetic alterations, indicating a positive feedback loop between fibroblasts and abnormal ECM (Klingberg et al., 2013; Parker et al., 2014).

Although there are many studies on the pathogenesis of pulmonary fibrosis, there are still many unclear details. The emergence of a new view of the “gut–lung axis” provides a new idea for the study of pulmonary fibrosis pathogenesis agents.

## Microbiome alteration in pulmonary fibrosis

### Microbiome alteration in idiopathic pulmonary fibrosis

Idiopathic pulmonary fibrosis, is the most common form of pulmonary fibrosis. The earliest and most common research on the microorganisms in IPF patients relates to the change in the lung microbiome. Richter et al. (2009) found that compared with healthy people, the bacterial load in the lungs of IPF patients increased *Haemophilus, Streptococcus, Neisseria* and *Veronella* at the species level. Another experiment demonstrated that pulmonary bacteria may play a pathogenic role in acute exacerbations of IPF and that increased relative abundance of specific species (*Streptococcus* and *Staphylococcus*) at diagnosis may be a biomarker of rapid disease progression (Han et al., 2014). Moreover, Invernizzi et al. (2021) found an increased number of *Actinomycetes* and *Veillonella* in patients with IPF compared with other lung diseases, and a correlation with survival. Huang et al. (2017) analyzed specific microbial colonization of the respiratory tract and found that there was a reasonable mechanistic link between the bacterial community and fibroblast reactivity.

Because the feces of IPF patients can be affected by factors other than the disease itself, such as age, living habits, genetic background, and treatment regimens, it is difficult to study. To date, there are no relevant data on the gut microbiome of IPF patients, but a recent animal model study has been conducted. Gong et al. (2021) found that 412 microorganisms at the genus level and 26 metabolites changed synchronously in the two mouse models [bleomycin (BLM)-induced and silica-induced] with the same trend, and there were significant differences from the control group. The combination of seven microorganisms (*Alloprevotella*, *Dubosiella, Helicobacter, Olsenella, Parasutterella, Rikenella*, and *Rikenellaceae RC9 gut group*) and nine metabolites (trigonelline, betaine, cytosine, thymidine, ophocholine, taurocholate, adenine, deoxyadenosine, and deoxycytidine) selected was validated to distinguish pulmonary fibrosis from normal controls. Among these, *Dubosiella* was positively correlated with betaine, but negatively correlated with cytosine, adenine, deoxyadenosine, and deoxycytidine; *Rikenella* was positively correlated with cytosine, thymidine, ophocholine, adenine, deoxyadenosine, and deoxycytidine (Gong et al., 2021).

### Microbiota changes in pulmonary fibrosis associated with systemic sclerosis

Systemic sclerosis (SSc), is an immune-mediated rheumatic disease characterized by vascular lesions and fibrosis of the skin and internal organs, with a high incidence of severe pulmonary fibrosis (Denton and Khanna, 2017; Perelas et al., 2020). A previous study observed a decrease in the symbiotic gut microbiome (e.g., *Faecalibacterium* and *Clostridium*) in SSc patients and an increase in bacteria (e.g., *Fusobacterium* and γ*-Proteobacteria*). As the severity of the disease increased, *Bacteroides fragilis* gradually decreased, and *Fusobacteria* gradually increased. Interestingly, these SSc patients also had increased numbers of *Bifidobacterium* and *Lactobacillus* (Volkmann et al., 2016). In a clinical study, SSc patients with pulmonary fibrosis had a more severe gut microbiota imbalance than those without pulmonary fibrosis, and presented in the early stage of fibrosis, suggesting that changes in gut microbiota in susceptible people may have the potential to predict the development of fibrosis (Andréasson et al., 2016). In SSc skin and lung fibrosis mouse models immunized with dendritic cells loaded with topoisomerase I peptide, Mehta et al. (2017) found that an increase in the ratio of *Bacteroidetes/Firmicutes* by oral administration of streptomycin caused progression of pulmonary fibrosis. At present, the theory that the gut microbiome could ameliorate the pulmonary symptoms of SSc has been applied in clinical treatment trials. In a 16-week clinical trial of patients with SSc treated with fecal microbiota transplantation (FMT) using commercially available anaerobic cultivated human intestinal microbiota, lung function [based on the diffusing capacity of lung carbon monoxide (DLCO)] improved significantly, which also proved the effect of the gut microbiota on lung lesions in SSc patients (Fretheim et al., 2020).

### Silicosis-related gut microbiota changes

Silicosis is an occupation-related progressive pulmonary fibrosis resulting from prolonged inhalation of dust with high concentrations of free silica (Barnes et al., 2019). Zhou et al. (2019) found that *Firmicutes* and *Actinobacteria* in silicosis patients were reduced at the phylum level in stool samples compared with those of healthy subjects. At the genus level, the *Devosia, Clostridiales, Alloprevotella* and *Rikenellaceae_RC9* levels decreased, and the *Lachnospiraceae* and *Lachnoclostridium* levels increased. They also predicted that these bacteria were mainly involved in biological processes such as energy metabolism and transport, membrane structure and function, and gene expression (Zhou et al., 2019). The mechanism of microbiota in pulmonary fibrosis needs to be further verified. Similarly, changes in the microbiota were also found in animal models. Gong et al. (2021) found that at least 412 kinds of intestinal microbes at the genus level compared and 26 kinds of metabolites changed. Seven microorganisms and nine metabolites were associated with pulmonary fibrosis. Among them, the metabolite trigonelline was significantly increased (Gong et al., 2021), which has antioxidant, anti-inflammatory and cell-protective effects (Khalili et al., 2018).

### Radiation-induced lung fibrosis gut microbiome changes

Radiotherapy is an important treatment for cancer, but its therapeutic side effects cannot be ignored. For example, 10% to 30% of patients with lung or breast cancer who receive radiation therapy will develop radiation-induced pneumonitis, and they are likely to develop radiation-induced lung fibrosis (Fleckenstein et al., 2007; Chao et al., 2017). In mice with local chest irradiation, Chen et al. (2021) found that *Akkermansia, Desulfovibrio* and *Parasutterella* increased and *Rikenella* decreased at the genus level and that *Distasonis*, *Goldsteinii*, and *Rodentium* decreased at the species level. These changes were ameliorated after FMT (Chen et al., 2021).

All the above evidences indicate that the microbiota is altered in pulmonary fibrosis (Table 1). Although no specific mechanism of action of individual microbiota has been investigated, a growing body of literature has found that microbiota metabolites play an important role in pulmonary fibrosis, which will be discussed in detail next.

**TABLE 1:** Intestinal microflora detection experiment of pulmonary fibrosis.

The type of pulmonary fibrosis	Detection object	Compare object	The changes of gut microbiome	Results	Conclusion	References
Idiopathic pulmonary fibrosis	Bleomycin mouse models	Normal mouse	*Alloprevotella*, *Helicobacter*, *Rikenella*, *Rikenellaceae RC9* ↓ *Dubosiella*, *Olsenella*, *Parasutterella* ↑	The microflora of 412 genera were significantly different from that of the control group with 26 metabolites.	A link between the gut microbiome and pulmonary fibrosis was found in a mouse model and demonstrated that they could distinguish pulmonary fibrosis from normal controls.	Gong et al., 2021
Pulmonary fibers associated with SSc	SSc patients with pulmonary fibrosis	Health people	*Faecalibacterium, Clostridium, Bacteroides fragilis* ↓ *Fusobacterium, Bifidobacterium, Lactobacillus*,γ*-Proteobacteria* ↑	Dysbiosis occurred in 75% of patients, and was more severe in SSc patients with pulmonary fibrosis	It suggests that abnormal gut microbiome may contribute to the development of systemic inflammation and fibrosis in SSc.	Andréasson et al., 2016
	TOPOIA DCs induced SSc mice by oral streptomycin in early life	TOPOIA DCs induced SSc mice fed without streptomycin	The ratio of *Bacteroidetes/Firmicutes* ↑	Exacerbation of pulmonary fibrosis and dysregulation of pulmonary T cell response	Changes in the gut microbiome caused by streptomycin can exacerbate fibrosis in lung areas of SSc.	Mehta et al., 2017
	SSc patients with intestinal symptoms after fecal microbiome transplantation	SSc patients with intestinal symptoms before fecal microbiota transplantation	–	Lung function was effectively improved	Regulating gut microbiome of SSc patients can effectively improve lung function.	Fretheim et al., 2020
Silicosis	Silica mouse models	Normal mouse	*Alloprevotella, Helicobacter*, *Rikenella, Rikenellaceae RC9*↓ *Dubosiella*, *Olsenella*, *Parasutterella* ↑	The microflora of 412 genera were significantly different from that of the control group with 26 metabolites.	A link between the gut microbiome and pulmonary fibrosis was found in a mouse model and demonstrated that they could distinguish pulmonary fibrosis from normal controls.	Gong et al., 2021
	Silicosis patients	Health people	*Firmicutes, Actinobacteria* *Devosia*, *Clostridiales, Alloprevotella and Rikenellaceae RC9*↓ *Lachnospiraceae*, *Lachnoclostridium* ↑	The microbiome in the gut of silicosis patients changes.	It shows the relationship between silica-induced progressive pulmonary fibrosis and changes in intestinal microbial diversity in humans	Zhou et al., 2019

SSc, systemic sclerosis; TOPOIA DCs, topoisomerase I peptide-loaded dendritic cells.

## Metabolites of the gut microbiome

In recent years, a number of studies have shown that intestinal microbial metabolites, including amino acids, short-chain fatty acids (SCFAs), bile acids, and valproic acid, are involved in the process of pulmonary fibrosis through ECM accumulation, energy metabolism, epigenetics, immune regulation and other pathways (Chen et al., 2017; Bai et al., 2019; Fang et al., 2020). We will discuss these in more detail next (Table 2).

**TABLE 2:** Effects of gut microbiome metabolites in pulmonary fibrosis.

Metabolites	Variation trend	Effector	Effector mechanism	References
Glutamate	Increase	Collagen production	Glutamic–glutamine cycle involves in collagen production of myofibroblasts	Bernard et al., 2018; Hamanaka et al., 2019
		Fibroblasts apoptosis	Glutamine promotes anti-apoptosis of IPF fibroblasts through epigenetic regulation of apoptosis suppressor proteins	Bai et al., 2019
Arginine	Increase	Airway tension	NO produced by arginine metabolism is involved in regulating airway tension and affecting respiratory immune stress	Fu et al., 2020
		Collagen deposition	Proline from arginine metabolism is the rate-limiting substrate in collagen synthesis and is essential for collagen precipitation in pulmonary fibrosis	Niese et al., 2010; Roque and Romero, 2021
		Fibroblasts activation	Arginine depletion attenuates the proliferation, migration and invasion of fibroblasts, thereby slowing down pulmonary fibrosis	Li et al., 2021
		Immune imbalance	Arginine combined with norvaline can correct the imbalance of immune cells in BLM mice	Gao et al., 2019
		Macrophage activation	Arginine induces the increase of GSH and inhibits the release of various proinflammatory cytokines from the macrophage	Hnia et al., 2008
		Collagen degradation	Arginine inhibits the activation of NF-κB to reduce MMP-2 and MMP-9 activities	Wang et al., 2015
Tryptophan	Decrease	T cell immune response	Metabolic derivatives of tryptophan produced by IDO reduce T cell inflammation	Lou et al., 2019
		T cell differentiation	Tryptophan and its metabolites regulate the transcription of multiple genes to affect T cell differentiation	Rothhammer and Quintana, 2019; Takei et al., 2020
		Collagen generated	The metabolite 5-MTP reduces myofibroblasts aggregation, differentiation and collagen precipitation	Fang et al., 2020
Butyrate	-	Gene expression	Butyrate inhibits Thy-1 gene expression and pulmonary fibrosis by inhibiting HDAC activation	Zhu et al., 2016
		Fibroblasts activation	Butyrate inhibits histone 3 acetylation to affect fibroblasts activation and exert antifibrotic effect	Park et al., 2021
		TGF-β1 production	The combination of valproic acid and butyric acid reduces the amount of NF-κB entering the nucleus and the production of TGF-β1, thereby alleviating pulmonary fibrosis	Chen et al., 2006; Sakai and Tager, 2013
Bile acid	Increase	Collagen generated	Bile acid stimulates fibrotic mediators to activate TGF-β1/SMAD3 signaling pathway and bile acid receptor FXR or induce the activation of alveolar epithelial cells and lung fibroblasts	Chen et al., 2016, 2017
PSA	Increase	Inflammatory response	PSA through TLR2 induces Foxp3^+^ Treg to produce IL-10 and TGF-β2	Round and Mazmanian, 2010
Valproic	Decrease	Epithelial-to-mesenchymal transition	Valproic affects histone H3K27 acetylation to inhibit epithelial-mesenchymal transition	Noguchi et al., 2015

IPF, idiopathic pulmonary fibrosis; NO, nitric oxide; BLM, bleomycin; GSH, glutathione; NF-κB, B-cell nuclear factor κ; MMP, matrix metalloproteinase; IDO, indoleamine 2,3-dioxygenase-1; AhR, aryl hydrocarbon receptor; 5-MTP, 5-methoxytryptophan; HDAC, histone deacetylase; TGF-β, transforming growth factor-β; PSA, polysaccharide A; TLR2, Toll-like receptor 2; Treg, regulatory T cell; IL-10, interleukin-10.

### Amino acids

**FIGURE 1:**
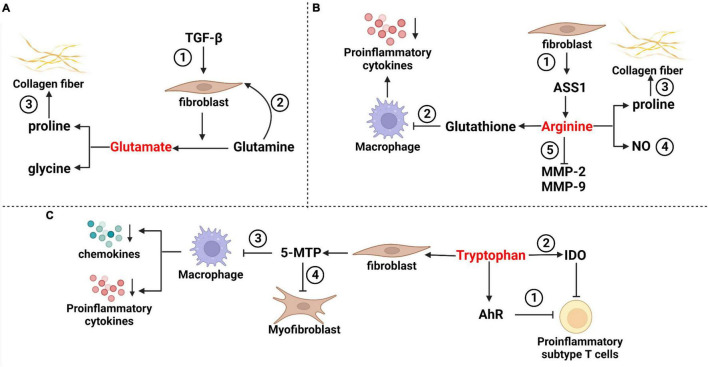
Mechanisms of partial amino acids in pulmonary fibrosis. **(A)** Glutamate in pulmonary fibrosis ➀ TGF-β stimulated fibroblasts, and increased the decomposition of glutamine and glutamate levels. ➁ Glutamine promoted anti-apoptosis of fibroblasts through epigenetic regulation of XIAP and survivin. ➂ Glutamate metabolite proline is a rate-limiting substrate for collagen formation in pulmonary fibrosis. **(B)** Arginine in pulmonary fibrosis ➀ Arginine has a variety of sources, including fibroblasts that can synthesize arginine via the ASS1 pathway often used in research experiments. ➁ Arginine can induce an increase in glutathione and inhibit the release of proinflammatory cytokines such as TNF-α, IL-1β and IL-6 from macrophages. ➂ Arginine metabolite proline is a rate-limiting substrate for collagen formation in pulmonary fibrosis. ➃ The NO isoforms of arginine metabolites include iNOS and cNOS, which have bidirectional effects on airway inflammation. ➄ Arginine inhibits the activation of NF-κB light chain enhancer and reduces the activities of matrix metalloproteinase-2 (MMP-2) and MMP-9 in fibrosis. **(C)** Tryptophan in pulmonary fibrosis ➀ Tryptophan acts with aryl hydrocarbon receptors to inhibit proinflammatory subsets of T cells. ➁ Tryptophan metabolites produced by IDO can indirectly reduce T-cell inflammation. ➂ Tryptophan is metabolized to 5-MTP in fibroblasts, which can inhibit macrophage activation and block the release of proinflammatory cytokines and chemokines. ➃ 5-MTP inhibits myofibroblast formation by interfering with the TGF-β/SMAD3 and PI3K/Akt pathways.

Amino acids, which can be obtained through the metabolic breakdown of proteins by the gut microbiome, are essential for cell survival, maintenance of normal function and proliferation (Chung et al., 2015; Ren et al., 2017; Cummings et al., 2018). IPF patients showed changes in amino acid metabolism, characterized by higher levels of proline, 4-hydroxyproline, alanine, valine, leucine, isoleucine, and lysine detected in lung tissue and exhaled breath (Gaugg et al., 2019). Many of these amino acids may function as signal transduction and regulatory molecules in diverse biological processes under various conditions, including gene expression, oxidative defense, and immune responses (Figure 1; Hatazawa et al., 2018; Glatzová et al., 2021; Jian et al., 2021).

#### Glutamate

Glutamate is a free amino acid found in protein (Tomé, 2018). There is a glutamate–glutamine cycle between glutamate and glutamine. That is, the carbon skeleton of glutamate is used for *de novo* synthesis of glutamine (Chen et al., 2018), while glutamine is converted to glutamate by glutaminase and then converted to the tricarboxylic acid cycle (TCA) metabolite α-ketoglutarate (α-KG) (Bai et al., 2019). Bernard et al. (2018) found that TGF-β stimulated the decomposition of glutamine by fibroblasts, resulting in an increase in α-KG and glutamate and a decrease in glutamine levels. When extracellular glutamine was lost, TGF-β-induced myofibroblast differentiation was prevented. Even after the removal of extracellular glutamine after fibroblast differentiation, the expression of the profibrotic markers fibronectin and hypoxia-inducible factor-1α was decreased, and TGF-β-induced metabolic reorganization was reversed (Bernard et al., 2018). Similarly, Hamanaka et al. (2019) explained that TGF-β-induced lung fibroblasts produced collagen, which requires glutamine and its conversion to glutamate through glutaminase. Furthermore, they confirmed that glutamate was converted to proline and glycine by the glutamate-consuming enzymes phosphoserine aminotransferase 1 and aldehyde dehydrogenase 18A1/D1-pyrroline-5-carboxylate synthetase (Hamanaka et al., 2019). In the lung tissues of human IPF patients, Bai et al. (2019) found that the deposition of glutamine decomposition products promoted anti-apoptosis of fibroblasts through epigenetic regulation of XIAP and survivin, members of the inhibitor of apoptosis protein family. All these studies suggested that the glutamate–glutamine cycle was a key component of fibroblast metabolic reprogramming.

#### Arginine

Arginine can be metabolized through two pathways, namely, the enzymes nitric oxide synthase (NOS) and arginase (Niese et al., 2010). The former product includes nitric oxide (NO), which can participate in the regulation of respiratory inflammation and airway tension and can affect the immune stress of the respiratory tract (Fu et al., 2020). The latter produces ornithine and proline via arginase and ornithine aminotransferase (Wu et al., 2021). It has been suggested that inhibition of arginase could reduce collagen deposition and improve BLM-induced pulmonary fibrosis (Roque and Romero, 2021). Niese et al. (2010) found that proline, which can be metabolized by arginine, is usually a rate-limiting substrate in collagen synthesis and is crucial for collagen precipitation in pulmonary fibrosis.

Li et al. (2021) found loss of arginine succinate synthase 1 (ASS1), a rate-limiting enzyme responsible for the biosynthesis of the endogenous semi-essential amino acid, arginine during the urea cycle, in fibroblasts from patients with IPF. They demonstrated that ASS1 gene deletion promoted the invasive and fibrotic potential of lung fibroblasts. Further studies suggested that exogenous arginine deprivation attenuates fibroblast proliferation, migration, and invasion, thereby protecting mice from BLM-induced pulmonary fibrosis (Li et al., 2021). Therefore, we speculate that ASS1 deficiency in fibroblasts during IPF development may represent a compensatory feedback regulatory mechanism to control endogenous arginine homeostasis.

Arginine also indirectly affects pulmonary fibrosis through immune cells. Gao et al. (2019) observed that combined treatment with arginine and norvaline significantly inhibited the increase in Tregs, γδT cells and Tregs/Th17 in BLM mice and ameliorated the decrease in Th17 cells, which inhibited the progression of pulmonary fibrosis. During the inflammation period, a small amount of the arginine metabolite NO is produced by metabolism in response to calcium-independent inducible nitric oxide synthase (iNOS) activated by Th1 cytokines for immune regulation and remodeling (Bronte and Zanovello, 2005). NO also dilates blood vessels and suppresses inflammation when produced in large amounts catalyzed by calcium-dependent cNOS. The arginine metabolite NO plays a bidirectional regulatory role in pulmonary fibrosis (Maarsingh et al., 2008). Arginine could induce an increase in glutathione (GSH) and inhibit the release of proinflammatory cytokines such as TNF-α, IL-1β, and interleukin-6 by macrophages (Wang et al., 2015). Arginine inhibited the activation of the NF-κB light chain enhancer regulating the activity of matrix metalloproteinase-2 (MMP-2) and MMP-9 (Hnia et al., 2008).

In general, arginine may contribute to the formation of pulmonary fibrosis by acting as a substrate for collagen deposition in pulmonary fibrosis or regulating immune disorders. However, other studies have found that NO metabolized by arginine has a bidirectional regulatory effect in pulmonary fibrosis. The mechanism of arginine’s role in pulmonary fibrosis has not been fully confirmed, and the bidirectional regulatory effect of arginine has not been fully explained, which will be investigated in future studies.

#### Tryptophan

Tryptophan is metabolized by indoleamine 2,3-dioxygenase-1 (IDO) to produce kynurenine derivatives in inflammatory tissues (Sittig et al., 2021). Reducing tryptophan levels induced Treg polarization directly (Yue et al., 2015) or, through its IDO derivative, reduced T-cell inflammation (Lou et al., 2019). In the gut, various tryptophan metabolites affect immune homeostasis by interacting with aryl hydrocarbon receptor (AhR), which is expressed in immune cells and regulates the transcription of multiple genes (Rothhammer and Quintana, 2019). In a BLM-induced mouse model, Takei et al. (2020) found that Tregs were increased and proinflammatory T-cell subsets were inhibited by stimulation of AhR, and pulmonary fibrosis was reduced compared with the control group. Other tryptophan metabolites also regulate lung inflammation and fibroblasts. Fibroblasts metabolize tryptophan to release 5-methoxytryptophan (5-MTP), which inhibits macrophage activation, and blocks the release of proinflammatory cytokines and chemokines, and the expression of COX2 in macrophages (Wang et al., 2016). Fang et al. (2020) reported that 5-MTP reduced BLM-induced collagen deposition, myofibroblast accumulation, and alveolar structure destruction in mouse models. *In vitro* experiments, confirmed that 5-MTP inhibited the differentiation of fibroblasts into myofibroblasts and slowed the progression of pulmonary fibrosis by disrupting the TGF-β/Smad3 and PI3K/Akt pathways (Fang et al., 2020). Niacin, a tryptophan gut microbiota metabolite, can induce the differentiation of Treg cells and IL-10-producing T cells and can promote the anti-inflammatory properties of colonic macrophages and dendritic cells (Singh et al., 2014). Tryptophan adjusts pulmonary fibrosis progression through its various derivatives of systemic immune regulation and its influence on pulmonary fibrosis.

### Short-chain fatty acids

Short-chain fatty acids are metabolized by the microbiota in the cecum and colon based on dietary fibrosis and proteins and peptides (Macfarlane and Macfarlane, 2012). Different gut microbes produce different types and proportions of SCFAs (Yang and Rose, 2014; Mirkoviæ et al., 2015). SCFAs can enter the systemic circulation and directly affect the function and metabolism of abenteric organs and tissues, such as the lungs, liver and skeletal muscle tissue (Boets et al., 2017). According to the literature, pulmonary SCFA levels are 370 times higher on average than those in blood (Segal et al., 2017). Ghorbani et al. (2015) found that high concentrations of SCFAs reduced alveolar epithelial cell line (A549) proliferation. SCFAs (mainly propionic acid and butyric acid) can completely restore respiratory epithelial barrier dysfunction caused by allergens or IL-4/IL-13 by improving the expression of tight junction proteins or inhibiting the MAPK pathway (Richards et al., 2020).

Zhu’s team, in a mouse model of pulmonary fibrosis induced by lipopolysaccharide (LPS), demonstrated that butyrate pretreatment could inhibit ThY-1 gene expression and pulmonary fibrosis by inhibiting histone deacetylase (HDAC) activation and histone H4 deacetylation (Zhu et al., 2016). Park et al. (2021) observed that sodium butyrate treatment attenuated BLM-induced skin and lung fibrosis in a BLM-induced mouse model. They found that sodium butyrate modulates macrophage differentiation in mesenteric lymph nodes and bronchoalveolar lavage cells of model mice, and inhibits TGF-β reactive proinflammatory expression by increasing histone 3 acetylation, thereby showing indirect and direct antifibrotic effects on fibroblasts (Park et al., 2021). It has been reported that the combined action of valproic acid and butyric acid can reduce the amount of nuclear factor kappa B (NF-κB) entering the nucleus and the production of TGF-β1 by inhibiting threonine kinase (Akt)/protein kinase B gene expression, thereby alleviating pulmonary fibrosis (Chen et al., 2006; Sakai and Tager, 2013). At present, there are few studies on SCFAs, and the role of different types of SCFAs in different types of pulmonary fibrosis needs to be further explored in the future.

### Other metabolites

At present, with the development of metabolomics, an increasing number of relationships between microbial metabolites and pulmonary fibrosis have been discovered. For example, Chen et al. (2016) reported that bile acids induced the activation of alveolar epithelial cells and pulmonary fibroblasts *in vitro*. Repeated inhalation of bile acid has been shown to cause pulmonary fibrosis in a mouse model, showing significant pathological changes in pulmonary fibrosis, enhanced expression of type I collagen, and an increased number of myofibroblasts. It was further observed that the main mechanism of its action was to stimulate fibrosis mediators and activate the TGF-β1/Smad3 signaling pathway and bile acid receptor FXR to promote fibrosis (Chen et al., 2017). Polysaccharide A (PSA) produced by the human symbiont Bacteroides fragilis can induce Foxp3^+^ Tregs to produce IL-10 and TGF-β2 through Toll-like receptor 2 (TLR2) expression, thereby regulating inflammation and alleviating the progression of pulmonary fibrosis. PSA can also induce the transformation of FoxP3^–^ Tregs into Foxp3^+^ Tregs expressing IL-10, which may be one of the mechanisms of PSA’s anti-inflammatory properties (Round and Mazmanian, 2010). In addition, Noguchi et al. (2015) found *in vitro* that valproic acid can partially inhibit TGF-β-induced epithelial-to-mesenchymal transition by improving histone H3K27 acetylation reduction, thus directly alleviating pulmonary fibrosis.

## Conclusion

The gut microbiome of patients/models with pulmonary fibrosis changed compared to healthy controls, although the changes in the microbiome of different types of pulmonary fibrosis were not consistent. The gut microbiome could participate in the process of pulmonary fibrosis through metabolites. For example, amino acid metabolism regulates fibroblasts to affect migration, transformation and collagen deposition. Amino acid metabolism can also regulate macrophage and T-cell activity to participate in proinflammatory or profibrotic processes. Butyrate increases histone acetylation by inhibiting HDAC activation, thereby inhibiting TGF-β and fibroblast expression. Bile acids activate the TGF-β1/Smad3 signaling pathway to induce the activation of alveolar epithelial cells and lung fibroblasts. At present, there are few studies on the single species/genus microbiome, and its specific mechanism in pulmonary fibrosis is not clear. In the future, in addition to the phenomenon of the changes in the gut microbiome and metabolites in pulmonary fibrosis, more research should be performed on their specific participation mechanisms. At present, the results of the gut microbiome and metabolites have been reported for clinical application. In the future, a deeper understanding of the association of the gut microbiome and metabolites with pulmonary fibrosis could provide a basis for finding diagnostic, therapeutic and prognostic targets.

## Author contributions

YlW and YhL wrote the review. CyT and YL contributed to the theme and structure of the review. YZ, JW, LC, XpL, and TW contributed to the literature search and summary. CyT, YL, and YbL made important modifications to important intellectual content. All authors read and approved the final manuscript.
